# From Shamans to Priests of Sekhmet: A Review of the Literature in Search for the Origins of Doctors in Ancient Egypt

**DOI:** 10.7759/cureus.67195

**Published:** 2024-08-19

**Authors:** Reinaldo B Bestetti, Rosemary F Daniel, Tufik M Geleilete, Ana Luiza N Almeida

**Affiliations:** 1 Medicine, University of Ribeirão Preto, Ribeirão Preto, BRA

**Keywords:** medicine history, history of medical sciences, physicians history, religion and medicine, shamanism, ancient egypt

## Abstract

This review suggests that shamans were in charge of the healing process in pre-dynastic Egypt. After the unification of Lower and Upper Egypt by Narmer in 3100 BC, shamans evolved into the Sem priests, who were responsible for the king’s health. With the change in Egyptian religion in the fourth dynasty (2613-2494 BC), Ra, the sun god, was revered as the supreme power, replacing the king. The emergence of mass festivities to celebrate Ra led to the priests of Sekhmet in the fifth dynasty (2494-2345 BC) checking the sanitation of bull meat that was provided to the populace in an attempt to avoid infectious epidemics. This seems to be the first recognition that disease might be transmitted from animals to humans. They used medical folklore, incantations, spells, and charms available at the House of Life, previously used by the lector priest. By 2487 BC, the first medical curative procedure was performed by Ni-Ankh-Sekhmet who cured the bleeding of a king's nose.

## Introduction and background

Introduction

The healing process of diseases affecting human beings dates back to immemorial times. Due to the capacity of humans to ascribe powers to natural phenomena (the so-called animism), disease was thought to be the consequence of malign spirits that inhabited the supernatural world [1].

Some people of the ancient communities were believed to communicate with such spirits and to ask, among other things, for healing the sick person. They were called shamans. Apart from their capacity to induce a placebo effect on the healing process, shamans may have learned some healing with animals in nature [2]. Such knowledge would have been transmitted from one generation to another orally.

The main characteristic of shamanistic activity is the trance state, which would allow shamans to enter the supernatural world, and ask gods for community well-being and healing disease people. Shamans would be guided in the supernatural world by spirit helpers, especially birds [3]. However, shamans might also perform divination and serve as advisors to a clan chief [4].

Healing processes, magical, divination, propitiation, and empiric use of plants have been seen in the Near East (mainly in Mesopotamia), Africa, and Egypt in the prehistory period. However, the exact place and period in which doctors emerged are still debatable. Accordingly, this review aims to provide an overview of the possible origins of doctors in ancient Egypt.

Methods

**Figure 1:**
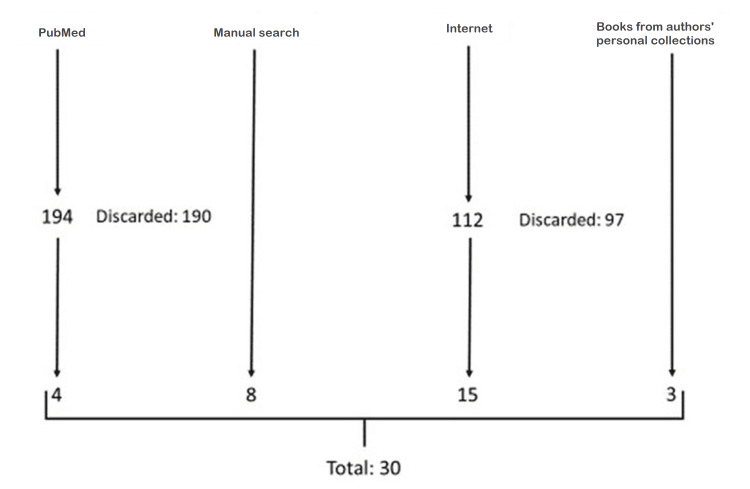
Search strategy for included literature

We did a search in PubMed without any language restriction using the Medical Subject Headings (MeSH) terms Physicians/history and Egypt, ancient; Physicians and ancient Egypt; doctors and ancient Egypt. A total of 194 papers were retrieved. All were read by title and by abstract. Only four papers were included in the study because they were concerned with the paper’s objective [4-7]. A manual search for the references of retrieved papers potentially useful for the study was performed, and of these, eight were included [1-3,8-12]. Furthermore, a search on the Internet for old books as well as old papers, not usually found in the PubMed database, was also performed because this could provide more information on the subject. A total of 112 papers/old books were retrieved and of these, 15 were included [13-27]. A manual search for the references found in such books and papers was also carried out. Three were included from the authors' personal collections were also included [28-30]. Figure 1 illustrates the search strategy for working out this research.

## Review

The healing process in the pre-dynastic Egypt

From 8600 BC onwards, hunter-gatherers and herders colonized the Nabta Playa region in the south desert of Egypt. These people are believed to have brought with them tamed cattle and pottery from other parts of Africa. They processed harvested wild plant foods and hunted local wild animals. Milk and blood from the cattle were used for food, but meat was not. They settled in the area during two millennia when climate changes forced them to search for other areas with abundant fresh water. Healing practices were unknown [28].

By 6500 BC, a community with subsistence practices of hunting, gathering, fishing, and small-scale cereal production developed on the edges of Lake Qarum in the Fayum region in Middle Egypt. Animal husbandry was also important for the subsistence of such people, and cereal production was practiced on a communal basis [28].

Almost nothing is known regarding cults carried out by such communities. Ethnographical studies have suggested that spiritual leaders of hunter-gatherer societies were usually shamans, who were also responsible for the healing process [3]. In this sense, a figurine comprising a cylindrical head with holes where feathers could be applied, mimicking human hair and beard, fixed in a wood body, suggests shamanistic activity [28]. In the pre-dynastic period, Egyptian shamans appear to have originated from the Near East or Africa [4].

The Badarian Culture

By 4500 BC, a community comprising agropastoral people developed in the region of Maghar Dendera, in Upper Egypt, giving birth to the Badarian culture. This culture was the first to have agriculture as the main mode of subsistence in southern Egypt. Badarian graves show evidence of social hierarchy. Fishing was very important, but hunting wild animals was not. People lived in small villages [28].

Priests appeared in societies where the main mode of subsistence was agriculture, like that of the Badarian region. However, the priests’ activities were exceptionally associated with healing at that time; actually, priests were responsible for agricultural rites [3]. Clay figurines of women used in cults associated with rebirth in the afterlife have been found in the tombs in the Badarian region. This suggests shamanistic activity. Evidence of healing cults was not found in the Badarian culture [1].

The Naqada Culture

From 4000 to 3600 AC, the Naqada I culture replaced the Badarian culture in the Maghar Dendera. The economy of this culture was characterized by domesticated animals, including pigs, fishing, small-scale cereal production, and fruits [29]. Female clay figurines were much more frequently found along with figurines of bearded men, supposedly used in the shamans’ magical activities. Each village had an animal deity identified with a clan [13].

In addition, the Naqada I culture showed well-healed fractures (only possible with the use of splints) in mummified remains [14], and the use of the malachite to treat eye disease [5]. It is highly likely that shamans were behind such empiric treatments, as a complex surgical procedure (limb amputation) was carried out 31,000 years ago in Asia, certainly by a shaman [8].

From 3600 to 3200 BC, the Naqada II culture appeared. People now lived in towns, not in villages. Because of the mastering of agriculture and irrigation, sedentism emerged and people no longer depended on hunting and gathering; however, animal husbandry was still important for subsistence. The increase of copper use, and the introduction of silver and gold, made this culture very wealthy [29].

The increased social complexity, inequality, and wealth as attested to by funerary evidence [29] might have given rise to the appearance of formal leader-rulers to manage intragroup and intergroup conflicts, based on either despotism or democracy. Hereditary chiefdom, supported by shamans, formed the basis of a ruling elite, the political power at the hand of the chief and the religious power at the hand of shamans [9]. Rulers were considered to be representative of gods [9].

The Nakada II people introduced spirits in the form of birds and reptiles [13]. As the society was growing in population, there came about increased complexity, inequality, social violence, the need to establish defenses, and the need for prosocial behavior to keep the clan cohesive, which led to the appearance of high gods i.e., those who could punish people for undesirable behavior and protect the community against evil forces [10]. In this way, spirits became gods over time.

Gods were believed to incarnate in animals; Horus and Thoth are examples of ancient gods of the Egyptian pre-dynastic period. A temple dedicated to Horus, the god associated with the falcon, dated 3400 BC, was found at Hierakonpolis. It is considered the oldest temple in Egypt. Much less is known about Thoth at that time. Thoth had a primitive shrine at Hierakonpolis at the end of the pre-dynastic period [15], and it was associated with healing later on. Horus was represented as a man with a falcon head and had no association with the healing process, whereas Thoth was shown as a man with an ibis head, and was associated with the healing process [16].

In a painted tomb found in Hierakonpolis by 3400 BC, votive figurines and stone artifacts show two men holding a Heka (magical sceptre) in a swaggering gesture, and another one using a leopard skin, thus suggesting shamanic activity [17]. In a time when writing had not yet been invented, this seems to be the only available evidence for shaman activity in that region.

From 3200 BC to 3100 BC, the Naqada III replaced the Naqada II culture. One important triumph of this culture was the invention of the first hieroglyphs. This allowed rulers to manufacture cylindric seals, thus reinforcing the ruler's power, as well as to identify state goods, allowing tight control of the economy by the king. Furthermore, there was an expansion of trade with cities of the eastern Mediterranean via the expansion of the culture to northern Egypt, which became a center of craftsmanship [18].

Rulers, who already were the representatives of gods as mentioned earlier, now considered themselves super-humans. Because of this, they associated themselves with gods in the late pre-dynastic period. This paved the way for the concentration of economic, political, and military power at the rulers’ hands. High-status burials suggest the presence of an economic elite [9].

Little is known regarding healing practices in pre-dynastic Egypt. The ibis has been shown in a bone label attached to a container found at Abydos circa 3300 BC [19], suggesting that a cult of Toth may also have existed there [16]. A study carried out with the healers of Agar Dinka in South Sudan, a traditional Nilotic agropastoral people with life conditions similar to that of the pre-dynastic period, may shed some light on ancient healing [11]. The Nilotic people comprise several clans; each clan has 600 inhabitants ruled by a chief, and healers, magicians, and exorcists practice healing. They revered a sacred cow, which was dedicated to a spirit or a deceased ancestor. A ritual was conducted by a healer, consistent with the transition from shaman to healer, in that the healer does not enter a trance state to healing [3]. The goal of the ritual was to prevent the sudden death of the cattle, and it was performed in shrines erected in the cattle camp. This is reminiscent of an incipient healing cult [11].

Healing deities, therefore, have not been found in predynastic Egypt [12], and the healing activity might have continued to be practiced by shamans. In fact, the body of a middle-aged woman along with paraphernalia consistent with shaman practice from the Egyptian pre-dynastic period has been found in a cemetery at Hierakonpolis [20].

Waterfowl, including the ibis, were considered spiritual helpers of shamans because it was believed that they could guide the shaman in the supernatural world. Ibis was revered because it was surmised to travel through the three tiers of the world (water, earth, and air). Actually, in a 9800 BC settlement in the Levant, many remains of ibis were found in a place supposed to be the center of shaman activity [21]. In the Neolithic, ibis was believed to be responsible for the formation of the cosmic egg, from which originated the world. Objects suggesting ibis deities have been found in the Egyptian pre-dynastic period. With the increase in the socioeconomic complexity of the pre-dynastic society, over time, shamans evolved into priests [3].

The appearance of centralized authority strengthened the power of priests who became the intermediary between gods and community without entering a trance state. Priests now had high socioeconomic status, as well as the responsibility of conducting political, legislative, judicial, and military activities [3]. Finally, the rulers became priest-kings [9].

The Egyptian early dynastic period

It is well known that by 3100 BC, Lower and Upper Egypt were united, thus giving rise to the dynastic period. This encompassed a region of about 1000 km from the Delta of Nile to Aswan. The central power was at Memphis. The king’s power was administered by ancient written stelae associated with the state gods. Palaces were built at Buto in the north and at Hierakonpolis in the south, and a temple with huge limestone figures dedicated to a fertility god (probably Min) was found at Koptos in the south. The surplus on agriculture controlled by the state led to Egyptian wealth in the dynastic period. The king, then, became a king-god. Shrines were frequently built in king tombs [18].

It was surmised that the king received the instructions directly from gods, and he was responsible for providing for the populace, including the healing activities. It was also pertinent to the king to supervise the activities at temples to appease gods, as may be the case in a primitive shrine of Toth at Hierakonpolis in 3100 BC [15,16].

The king chose the priests, the people who could get in touch with the gods, and took care of their daily needs [22]. Moreover, as a spiritual leader, the king could take part in magical healing [6], as previously practiced by shamans. In the first (3100-2890 BC) and second dynasties (2890-2686 BC), the kings were responsible for incipient public health. Medical texts likely appeared during this time [4]. In support of this, the Eber papyrus, which is dated 1600 BC, refers to King Deng, who ruled Egypt by 3000 BC [9].

According to Manetho, an Egyptian priest who lived in the fourth century BC, King Athotis of the first dynasty (by 3050 BC) practiced healing and wrote anatomical records [15]. However, no inscription in the tombs or even early papyruses has proved this statement. On the other hand, it is not unlikely that a king may have written something about anatomy. In fact, an alabaster bowl with the name of Athotis depicts the heart and six blood vessels leaving off the organ [6]. Furthermore, it has been suggested that kings practiced healing because they were acquainted with medical lore [15].

Priesthood was a well-hierarchized structure with different kinds of priests who had different duties [23]. The priest who was more related to the origins of the doctors was the Sem priest. The Sem priest wore leopard skin over the shoulders, like shamans did before, and practiced shamanism to cure. At first, during the first to the third dynasty (2686-2613 BC), the Sem priests were the king’s sons. Their responsibility was to take care of the purity of the king's food, nutrition, and hygiene, thus allowing the king to remain strong and healthy [4].

Apart from the impact on the king's health, the ritual of purity was believed to appease the gods; any imperfection in the rituals could have offended the gods, who then might have abandoned Egypt. In addition, the Sem priests also organized and commanded expeditions to the desert to mine copper, and it is believed that they provided occupational hygiene and some help with emergency health problems for mine workers. Finally, they were asked to invoke (in 3100 BC) the deity Serget, one of the oldest divinities associated with protection against scorpion envenomation, very common in ancient Egypt [4].

The lector priest appeared in 2890 BC [4], and practiced healing with magic, reminiscent of shaman activity [15]. As the name suggests, the lector priest was able to read the early papyruses at the House of Life, a place that functioned like a scriptorium but in which papyrus, especially those related to magic, could be read [15]. By the second dynasty (2890-2686 BC), it is believed that the lector priest might have transmitted written knowledge, as the majority of Egyptian people were illiterate. In addition, the lector priest could also take part in the king's cleanliness and in the inspection of the king's food every morning in order that the king could be presented as healthy and capable of acting as the reincarnation of the Sun God. This lasted until the fourth dynasty (2613-2494 BC) [4].

By the fourth dynasty (2613-2494 BC), however, there was a marked change in the Egyptian religion, when the concept of astronomically oriented stars was abandoned, ideas were centered on the Sun God to the point that the king was replaced by an eternal power manifested by the Sun God Ra. Furthermore, the kings of the fourth dynasty incorporated the title “Son of Ra” in their name. Finally, each king built a temple dedicated to Ra near his tomb [30]. The priests of Ra at Heliopolis may have fostered this change according to their personal interests [15]. Therefore, the care of the Sun God became more important than the care of the king.

The Sem priests then became responsible for mortuary rites. There is some evidence that the Sem priests entered the trance state in mortuary rites to find the soul of the deceased in the spirit world [24]. In the Old Kingdom (from the fourth to the fifth dynasty, 2613-2181 BC), high duties at court were ascribed to people outside the royal family, suggesting the start of reduced king power [30].

**Figure 2:**
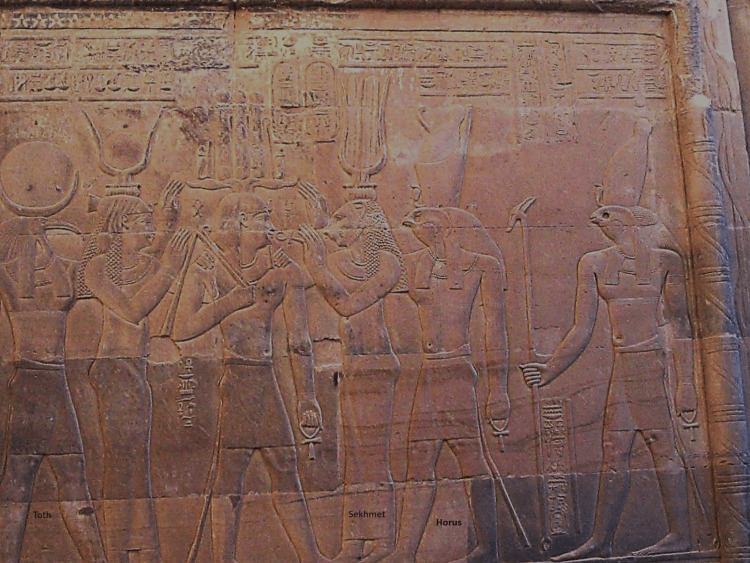
Sekhmet, with the head of a lioness in a woman's body, depicted on a wall relief in the Temple of Kom Ombo, Egypt Image Source: Authors

The food purity ritual, which had been exclusive to the king, now became important for public health in view of the several festivals devoted to the Sun God in which a lot of bulls were sacrificed for mass feeding. This allowed the appearance of the priests of Sekhmet (Figure 2) in the fifth dynasty (2494-2345 BC), who had the responsibility of inspecting the killed bull for food safety [4]. Sekhmet was a god represented by a lioness's head and a woman's body. It was a deity associated with violence and pestilence. However, it was also associated with healing. The priests of Sekhmet, by appeasing the goddess, were believed to be able to practice healing, especially as bonesetters [15].

Very little is known about the types of healing that occurred in the early dynastic period before the appearance of doctors. Doctors were believed to have acquired the medical knowledge under priesthood at the House of Life [23], from medical folklore [18]. Furthermore, medical knowledge could also be passed down from father to son [25]. It has been claimed that medical papyruses were available at the Houses of Life by the second dynasty [4]. At the House of Life, the scribe priest looked after the sacred books [15] as well as the medical papyruses, but not for mathematics or mere literature [25]. Therefore, the appearance of writing seems to have been paramount for the surge of doctors.

**Figure 3:**
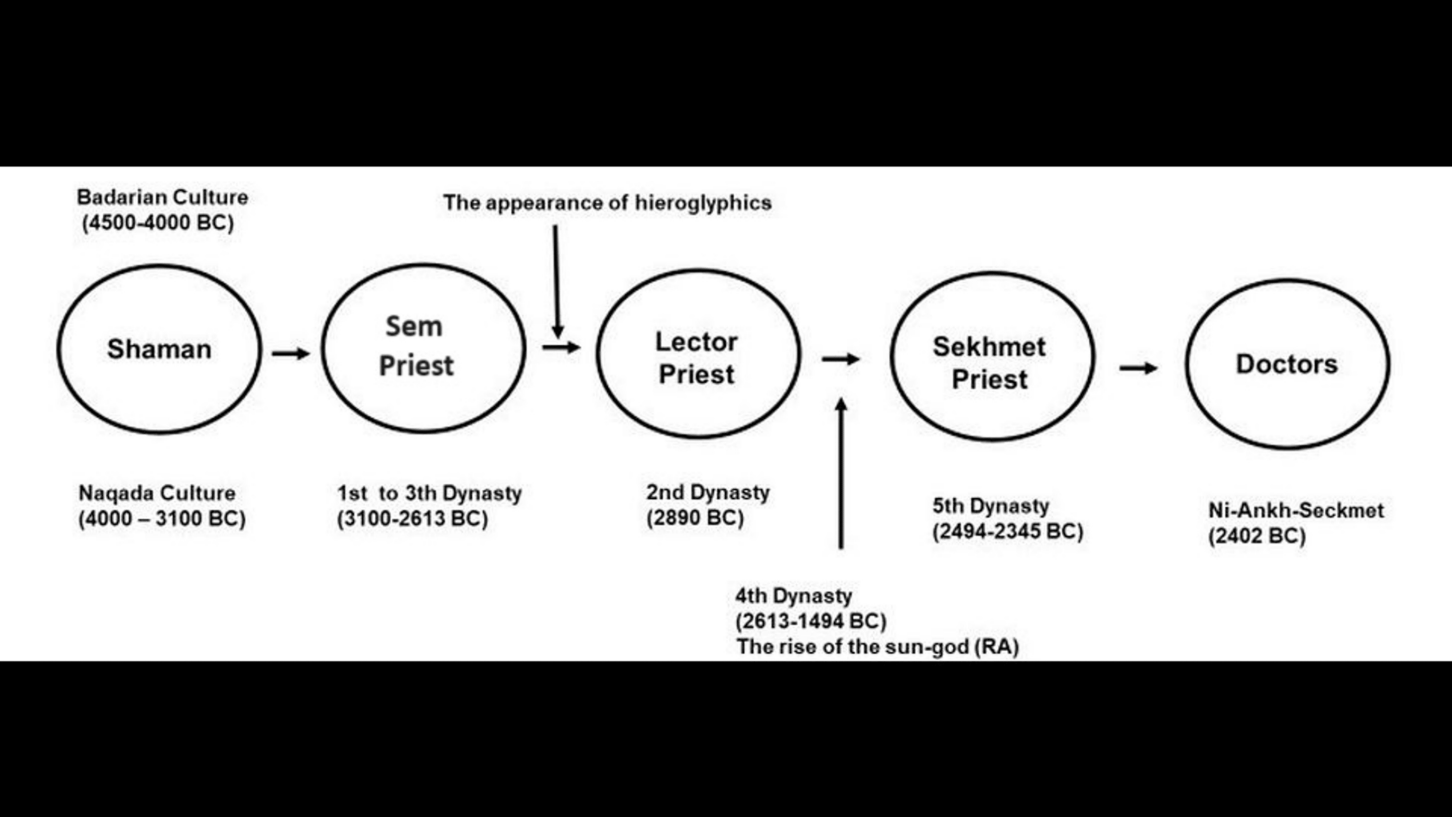
Timeline for the origin of doctors in ancient Egypt

It should be noted that in the Old Kingdom (2686-2181 BC), Thoth was revered by the priests as a god of wisdom, magic, scribes, and medicine [15], thus suggesting an evolution from shamans to priests to doctors. Sem priests and lector priests practiced healing using shaman skills in the early dynastic period [15]. With changes in religious concepts, Sem and lector priests abandoned the healing activities, and were assigned to mainly funeral activities. Since the official activities of all priests, in general, did not take place all through the year, they could carry out other activities (including healing) in the community in their free time [15]. Therefore, the priests of Sekhmet may be the origin of the professional doctors in the dynastic period [15,23-25]. Figure 3 illustrates the possible origins of doctors in ancient Egypt.

The early doctors

Hesy-Ra is believed to be the first recognized doctor in the world (2650 BC) at the time of King Djoser of the third dynasty (2686-2613 BC). There is a stela found in his tomb in Saqqara showing the title of Chief of Dentists and Doctors. However, details of his medical activities have not been found. It is open to debate, therefore, if Hesy-Ra was a real doctor or a priest in charge of supervision of healing activities at the king's court [22].

A contemporary of Hesy-Ra is Imhotep, the architect in charge of the construction of the oldest stone building in the world, the Pyramid of Saqqara. Imhotep was the chief of lector priests at Memphis. Since the lector priests were associated with funeral rites, this might have given Imhotep the opportunity to see postmortem examinations. In addition, he was an astrologer to the priests of Ra in Heliopolis [26]. Therefore, Imhotep could take part in the process by which Ra was transformed into the main god of Egypt [30]. Nevertheless, there is no proof that Imhotep practised healing. The first reference to the Imhotep’s healing practice is in the 13th dynasty and another reference was made by the priest Manetho in the third century BC. Because Imhotep was deified as the god of medicine, similar to Asclepius in Greece, in the Ptolemaic period in Egypt (third century BC), it is conceivable that the confusion originated at that time. Clearly, there is no evidence that Imhotep was a doctor [22].

Peseshet is believed to be a female doctor based on inscriptions found in the tomb of Akhet-Hotep at the Giza pyramid during the fifth dynasty (2494-2345 BC). The inscription states that she was the overseer of doctors. However, this is very unlikely because Peseshet would have been the only woman doctor registered in history, and it is improbable that she was allowed to oversee healing activities made by men in ancient Egypt [22].

A recently discovered tomb of another doctor, Shepseskaf-Ankh, dated the fifth dynasty, during the rulers Nyuserre, Menkauhor, and Djedkare at Abusir shows that doctors also practiced beyond Saqqara. This doctor was the chief physician at the king's court and the chief of physicians of Lower and Upper Egypt. He also was the priest of the god Khnum, the god related to the House of Life (where medical texts were written and stored) and the House of Protection (the place in which children were born). Nonetheless, the kind of healing he practiced is shrouded in mystery [27].

The first physician who clearly practiced medicine in the world appears to be Ni-Ankh-Sekhmet, who lived during the time of King Sahura (2487-2475 BC) in the fifth dynasty. A false door in the Ni-Ankh-Sekhmet’s tomb in Saqqara clearly states that the doctor healed the king’s nosebleed. Therefore, Ni-Ankh-Sekhmet must be considered the first physician in the world to perform a curative procedure [7].

## Conclusions

This review suggests that doctors evolved from the Sem priests in the early dynastic period, who practiced shamanistic healing activities, and from the lector priests, who used magic in the healing process. Thanks to the change in Egyptian religion, the priests of Sekhmet became responsible for​​​​​​​ the healing process from the fifth dynasty onwards. Ankh-Sekhmet seems to have performed the first curative procedure around 2487 BC, also in the fifth dynasty. This appears to precede the appearance of Mesopotamian doctors by 400 years.
